# Silence and reduced echolocation during flight are associated with social behaviors in male hoary bats (*Lasiurus cinereus*)

**DOI:** 10.1038/s41598-021-97628-2

**Published:** 2021-09-20

**Authors:** Aaron J. Corcoran, Theodore J. Weller, Annalise Hopkins, Yossi Yovel

**Affiliations:** 1 Department of Biology, University of Colorado, Colorado Springs, 1420 Austin Bluffs Blvd, Colorado Springs, CO 80918 USA; 2 USDA Forest Service, Pacific Southwest Research Station, 1700 Bayview Dr., Arcata, CA 95521 USA; 3 School of Zoology, Tel Aviv University, P.O. Box 39040, 6997801 Tel Aviv, Israel

**Keywords:** Behavioural ecology, Ecophysiology

## Abstract

Bats are renowned for their sophisticated echolocation. However, recent research has indicated that bats may be less reliant on echolocation than has long been assumed. To test the hypothesis that bats reduce their use of echolocation to avoid eavesdropping by conspecifics, we deployed miniature tags that recorded ultrasound and accelerations on 10 wild hoary bats (*Lasiurus cinereus*) for one or two nights. This resulted in 997 10-s recordings. Bats switched between periods predominated by their typical high-intensity echolocation, or periods predominated by micro calls (unusually short, quiet calls), or no detectable calls (“silence”). Periods of high-intensity echolocation included high rates of feeding buzzes, whereas periods of micro calls and silence included high rates of social interactions with other bats. Bats switched back to high-intensity echolocation during actual social interactions. These data support the hypothesis that bats use reduced forms of echolocation and fly in silence to avoid eavesdropping from conspecifics, perhaps in the context of mating-related behavior. They also provide the strongest demonstration to date that bats fly for extended periods of time without the use of echolocation.

## Introduction

Echolocation allows bats to occupy diverse nocturnal niches, including being the predominant predator of nocturnal flying insects^1–4^. Insectivorous bats use highly specialized echolocation to orient in darkness and it has long been assumed that bats are reliant on biosonar for nocturnal orientation^5–7^. Cases of bats not using echolocation have been limited to periods of a few seconds or less when passively listening to sounds of prey^8,9^ or avoiding interference from conspecifics^10^. Bats may be under selective pressure to reduce the intensity of their emissions (i.e., “stealth echolocation”) to avoid eavesdropping by prey^11,12^.

Hoary bats (*Lasiurus cinereus*) provide an interesting test case for understanding the limitations of biosonar and the selective pressures driving acoustic behaviors. Hoary bats use high-intensity echolocation emissions typical of bats that hawk insects in the open^13^ (hereafter “high-intensity echolocation”). However, in a recent study, researchers used arrays of infrared cameras and calibrated ultrasonic microphones to show that hoary bats switch between high-intensity echolocation and two previously unknown acoustic behaviors: “micro calls” and flight in apparent silence^14^.

Hoary bats produce a wide range of echolocation calls that fall along a continuum of acoustic parameters^13^. In addition, bats alter their calls according to task. For example, as they approach prey, they increase echolocation pulse rates and frequency bandwidth while decreasing pulse durations until ending in a terminal phase of very short, rapid pulses known as a feeding buzz. However, micro calls appear to be a discrete category of echolocation with much shorter duration, higher frequency, and lower source level compared to high-intensity search and approach calls or feeding buzzes^14^. Each of these factors reduces the effective range of micro calls with no apparent benefit to the bat from a sensory perspective as bats were observed using micro calls in mostly open habitats. Therefore, it was argued that micro calls and apparent cases of silence are adaptations for reducing eavesdropping by conspecifics. Corcoran and Weller^14^ frequently observed hoary bats chasing conspecifics in the field and demonstrated that they were attracted to playbacks of high-intensity hoary bat echolocation. Micro calls and silence have thus far only been documented during the hoary bat’s mating period^15^, which also coincides with autumn migration. Based on this information, the authors proposed that micro calls and silence are stealth behaviors used to avoid detection by conspecifics during mating-related activities.

In the previous study, ground-based recording methods limited observation periods to only a few seconds at a time at three locations. Furthermore, it is possible that apparent cases of silence were simply a failure of the microphones to detect the low-intensity and directional micro calls. To overcome these limitations, we attached miniature (2.9 g) ultrasound and acceleration recording devices (“tags”;^16,17^) to wild, freely behaving male hoary bats (females were only rarely captured at our field location and were not available for tag deployments). Bats were captured at the same time of year (autumn) and location (Humboldt Redwoods State Park, California) as the previous study^14^, allowing us to compare results between on-board and ground-based recording methods.

Our primary hypothesis (H_1_) was that hoary bat switch from using high-intensity echolocation to use of micro calls and silence to avoid detection from conspecifics. Based on this hypothesis, we predicted that at longer time scales (many minutes), social interactions with conspecifics would be more common when bats are using micro calls and flying in silence and that at finer time scales (seconds), social interactions would be immediately preceded by micro calls or silence. We considered three alternate hypotheses that micro calls and silence are adaptations for avoiding detection by prey (H_2_), are employed during commuting in open space when echolocation is not needed for orientation (H_3_) and are used more frequently on clear moon-lit nights when vision could supplement echolocation (H_4_). Our results provide the strongest evidence to date that bats regularly forgo echolocation for extended periods in flight and that inconspicuous echolocation behaviors are aimed at avoiding detection by conspecifics.

## Results

We recovered 10 of 27 tags that were deployed on male hoary bats captured in Humboldt Redwoods State Park, California between September 21st, and October 7th, 2018. We recovered tags from bats tagged between September 28th and October 7th at distances ranging from 270 to 19 km from their release site. Five tags recorded data on one night and five others recorded for two nights. We obtained a total of 2241 recordings that each lasted 10 s, for a total of 373.5 min of recording. We used a combination of accelerometer data and low-frequency wind noise data to determine whether bats were flying in each recording (see Methods). This resulted in 997 recordings where bats were determined to be flying.

### Bats switch between high-intensity echolocation, micro calls, and silence

**Figure 1:**
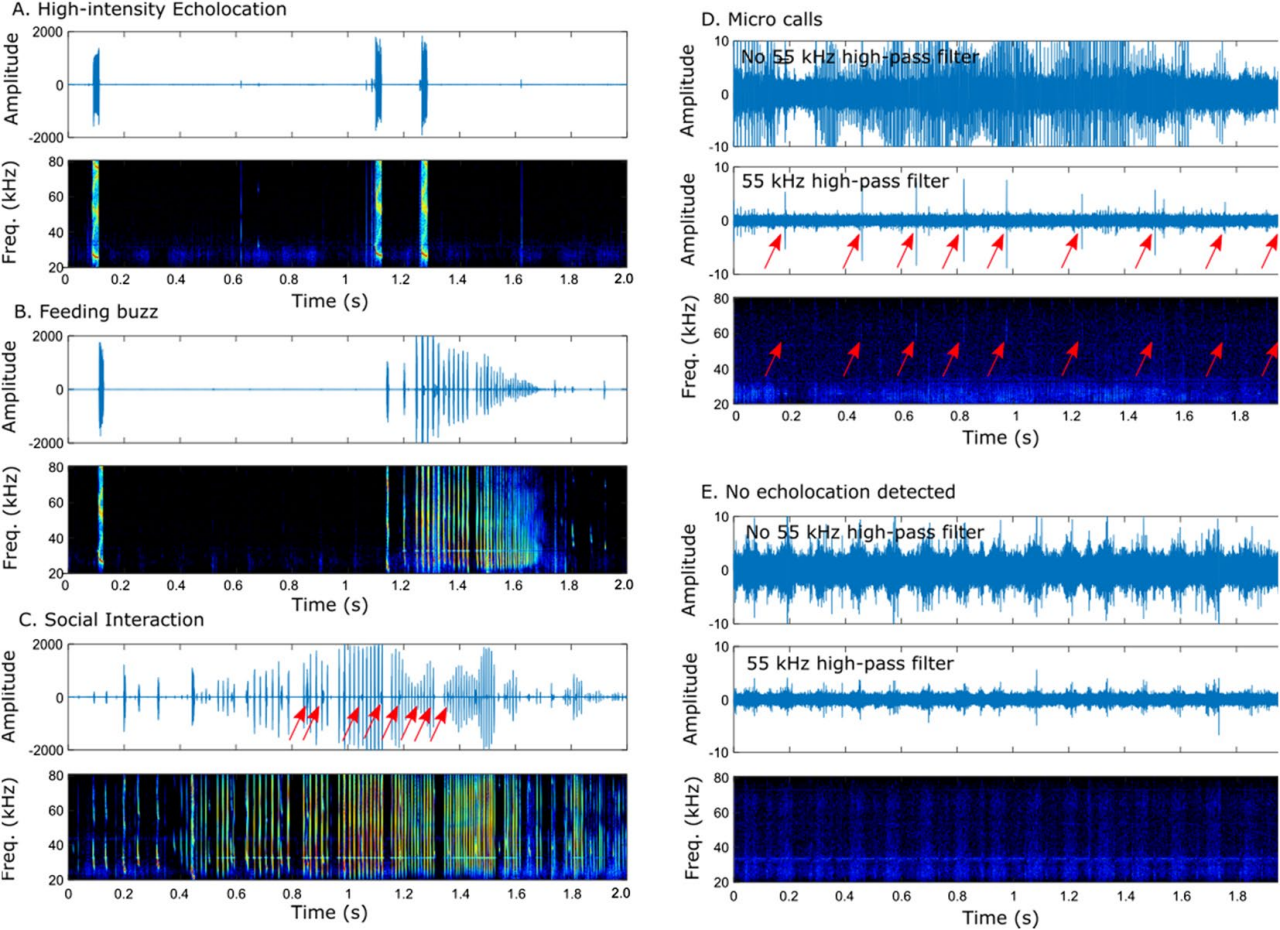
Example acoustic behaviors. Oscillograms (amplitude *vs.* time; top) and spectrograms (frequency *vs* time; bottom) are shown for clips of recordings to illustrate different acoustic behaviors. Two-second clips shown here were taken from longer 10-s recordings. A single oscillogram trace is shown for (**A**–**C**). Two oscillogram traces are shown for (**D**,**E**), one with and one without a 55-kHz high-pass filter, which removes low frequency wind noise and highlights the frequency range of micro calls. (**A**) High-intensity echolocation. A single echolocation pulse followed by an echolocation doublet. Note that these signals were overloaded on the microphone, resulting in spectrogram artefacts (**B**) Feeding buzz. A single echolocation call is followed by approximately 1-s pulse interval into a rapid feeding buzz. (**C**) Social interaction. Close inspection shows two individual bats—one calling at a very high rate and a second calling at a slightly lower rate. Some calls of the second bat are indicated by the red arrows (**D**) Micro calls. Red arrows indicate the locations of micro calls, which are evident in the oscillogram after applying a 55 kHz high-pass filter. Note that not all micro calls are visible in the spectrogram at the displayed resolution. (**E**) No detected echolocation. Note the temporal modulation of noise that occurs at approximately 8 Hz as a result of the bat’s wingbeats (also see Fig. S2). Also note the difference in the amplitude scale of oscillograms for (**A**–**C**) versus (**D**,**E**).

Each 10-s recording was classified by the predominant form of echolocation including high-intensity echolocation (721 out of 997 recordings; 72.3%; Fig. 1A; Supplementary Audio File 1), feeding buzzes (33 of 997 recordings; 3.3%; Fig. 1B; Supplementary Audio File 2), acoustic events where two bats produce calls at high repetition rates that is indicative of chasing behavior^14^; hereafter “social interactions” (33 of 997 recordings; 3.3%; Fig. 1C; Supplementary Audio File 3), micro calls (55 of 997 recordings; 5.5%; Fig. 1D), and files in which no echolocation calls were detected for at least five seconds; hereafter “silence” (155 of 997 recordings; 15.5%; Fig. 1E; Supplementary Audio File 4).

Echolocation calls from other bats (i.e., not the bat with the tag) were present in 67 of the above recordings (6.7%; See Supplementary Information). Echolocation behaviors indicative of chasing behavior were present in 33 (49.2%) of the recordings when other bats were present. Echolocation calls of other bats were present in 2.8% of sequences classified as high-intensity echolocation, 5.5% of micro call recordings, 6.5% of silence recordings, and 3.0% of feeding buzz recordings. Compared to micro and silence recordings, which were lumped for statistical analysis, high-intensity recordings had a significantly lower rate of other bats present (Fisher’s exact test; one-tailed distribution; *P* = 0.02). This indicates that bats using inconspicuous echolocation fly close to other bats more frequently than when they use high-intensity echolocation.

**Figure 2:**
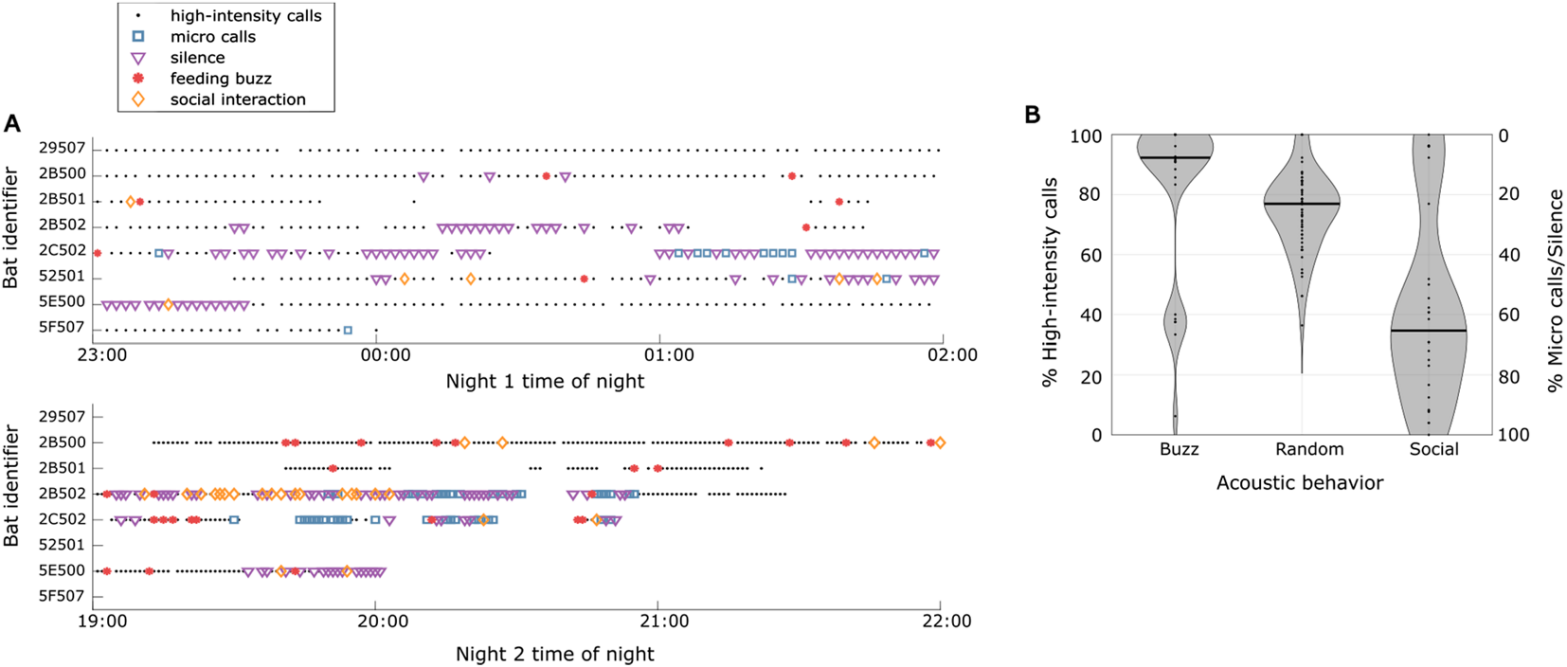
Acoustic behavioral patterns. (**A**) Time course of echolocation behaviors over the first and second nights of recording for eight individual hoary bats. Ten second data clips were recorded once every three minutes on the first night and once per minute on the second night. Data gaps reflect periods when bats were determined to not be flying. (**B**) Violin plot showing percent of recordings occurring within 15 min of different acoustic behaviors that are predominated by high-intensity calls *vs* micro calls or silence. Black dots show individual data points; grey areas indicate probability density functions. Note that feeding buzzes tend to occur more often during periods of high-intensity echolocation compared to if acoustic behaviors were randomized (“random”), whereas social interactions are more common when bats employ micro calls and silence.

The time course of acoustic behaviors across nights (Fig. 2) shows that bats switch between periods predominated by different types of echolocation behavior. For example, bat 5E500 begins the recording period on the first night where 10-s periods of silence were documented for a period of 26 min with a single chasing interaction. It then switches to using only high-intensity echolocation within documented 10-s periods over the following 2 h and 23 min. On the second night this bat begins the recording period with high-intensity echolocation and two feeding buzzes within recorded intervals over the first 31 min and then predominantly uses silence during recordings for the next 28 min. Relative use of different echolocation behaviors appears to vary among individuals (See Supplementary Information).

### Social interactions are associated with periods of micro calls and silence

To determine relationships between different acoustic events, we isolated each social interaction and feeding buzz and quantified the number of recordings occurring within 15 min (before or after) that were predominated by either high-intensity echolocation or reduced echolocation (micro calls or silence). Only 41.7% of recordings occurring within 15 min of social interactions involved high-intensity echolocation. This was significantly less than the 75.6% value that would occur by chance (permutation test; 10,000 randomizations; *P* < 0.0001; one such randomization is shown in Fig. 2B). In contrast, 84.4% of acoustic events recorded within 15 min of a feeding buzz had high-intensity echolocation (Fig. 2B). This was significantly higher than would occur by chance (permutation test; 10,000 randomizations; *P* < 0.0001). Therefore, social interactions with other bats were more likely to occur during periods when bats were either silent or making micro calls, whereas feeding buzzes were more likely to occur during periods of high-intensity echolocation. Note that social interactions themselves always involved two bats producing high-intensity echolocation calls (Fig. 1C).

Acoustic behavior immediately preceding social interactions *vs.* feeding buzzes also differed. Out of 24 recordings with chases starting > 3 s after the beginning of the recording, 13 had periods of silence or micro calls. In contrast, out of 25 recordings with feeding buzzes starting > 3 s after the beginning of the recording, none contained a > 3 s period of silence and only one contained micro calls. This was a highly significant difference (Fisher’s exact test; *P* < 0.0001). These data support the hypothesis (H_1_) that micro calls and silence are aimed at avoiding eavesdropping by conspecifics, but not the hypothesis (H_2_) that they are used for avoiding eavesdropping by prey.

### Bats using silence or micro calls exhibit higher degrees of maneuvering

**Figure 3:**
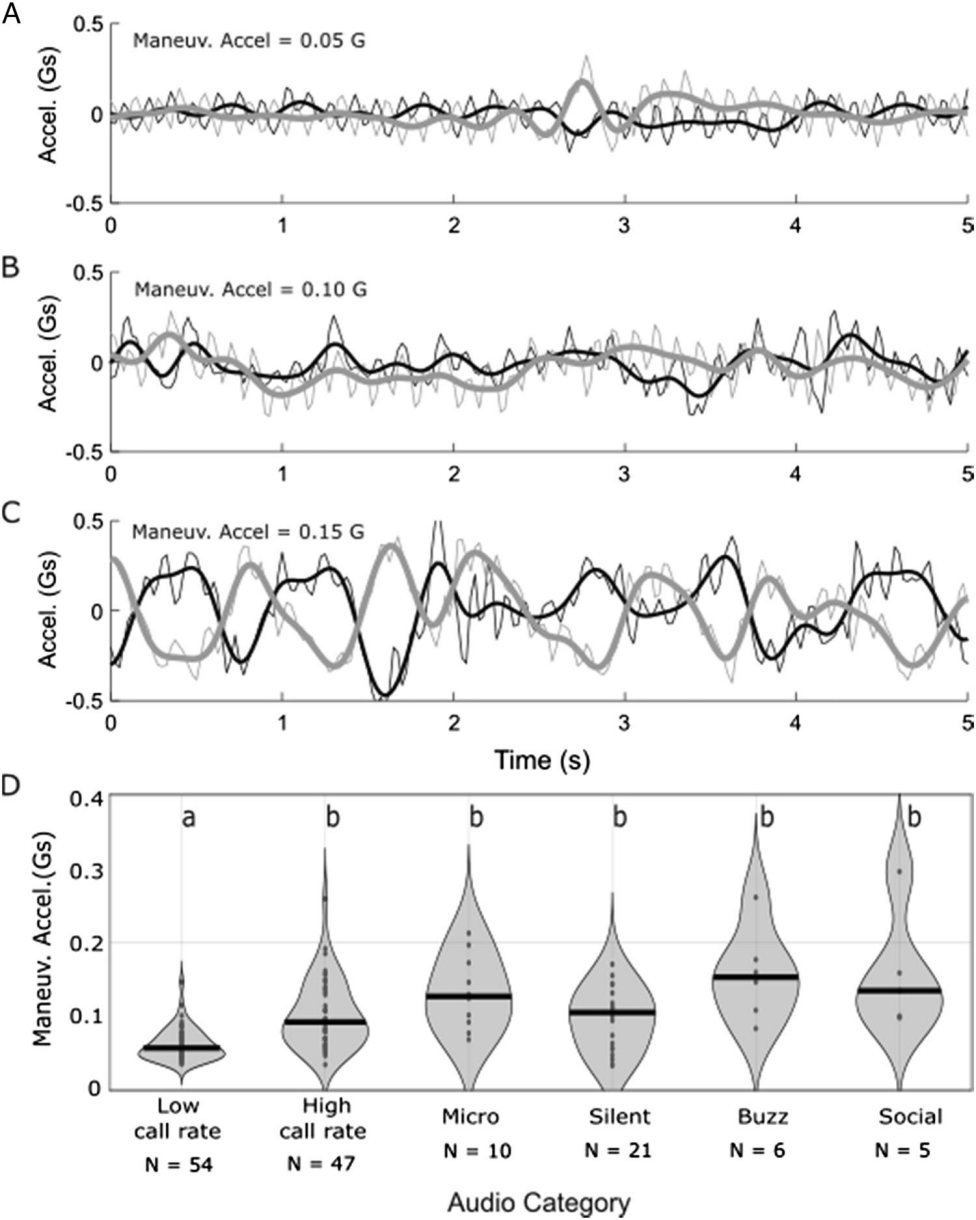
Flight maneuvering accelerations. Example acceleration traces show (**A**) low, (**B**) medium and (**C**) high degrees of maneuvering. Raw acceleration values are shown for lateral (thin black lines) and anterior–posterior (thin gray lines) axes. The dorsoventral axis was not used because it was dominated by wingbeat accelerations. Thick black and grey lines show accelerations with a 4 Hz low-pass filter that removes wingbeat frequency cycles. The root mean square of low pass filtered accelerations was taken as the “maneuvering acceleration” for each recording and traces with low, medium, and high values were chosen for illustration. (**D**) Violin plots show the probability density functions of maneuvering accelerations during different acoustic events. Thick black lines indicate median values and small black dots show individual values for each 10 s recording. Letters above call types indicate groupings with significantly different medians (see text for statistics). Note that sample sizes for acoustic events are lower here because not all acoustic recordings had accompanying accelerometer data (See Methods).

Using accelerometer data, we compared the degree of maneuvering between bats engaged in different acoustic behaviors (Fig. 3). Bats exhibited the lowest flight maneuvering levels when producing high-intensity echolocation calls with low calling rates (the median pulse interval value of 659 ms was used to differentiate “low” *vs.* “high” calling rates). Bats maneuvered more when making high-intensity echolocation with high calling rate (pulse interval < 659 ms), using micro calls, flying in silence, making feeding buzzes and during social interactions (Fig. 3; Kruskal–Wallis Test with Tukey post-hoc tests; X^2^ = 58.6; d.f. = 6; *P* = 8.6e−11). These data do not support the hypothesis (H_3_) that bats use micro calls and silence for straight commuting flight.

### Echolocation behavior was not associated with nightly light levels

The proportion of recordings per night where bats used high-intensity echolocation was not correlated with moon illumination (linear regression; N = 13 nights; F = 3.75; *P* = 0.08) or cloud cover (linear regression; N = 13 nights; F = 0.01; *P* = 0.92). While the relationship with moon illumination approached significance, the direction of the relationship was the opposite of what we had predicted (bats using more, not less high-intensity echolocation on well-lit nights). Cloud cover measurements were obtained from the nearest weather station, 25.5 km away from the site where bats were released. Due to the unpredictability of the marine fog layer, it is possible that weather conditions were different at the locations where bats occurred during the time of recording. Nevertheless, we found no evidence supporting the hypothesis (H_**4**_) that hoary bats regulate echolocation use depending on light availability.

## Discussion

We used on-board acoustic recording devices and accelerometers to document previously unknown detail regarding the acoustic, flight, and social behavior of hoary bats flying in the wild. The evidence we report supports our main hypothesis (H_1_) that hoary bats use micro calls and silence to avoid eavesdropping by conspecifics and resulting unwanted conspecific interactions.

### Micro calls and silence are associated with conspecific interactions

Acoustic recordings revealed that bats tend to fly using one of two primary sensory modes—they either produce high-intensity echolocation with occasional feeding buzzes or they make micro calls or fly in silence with occasional social interactions indicative of chasing behavior (Figs. 1, 2). This pattern is consistent at broad time intervals of 15 min and within 10-s recordings. These data support our primary hypothesis (H_1_) that silence and micro calls are associated with conspecific interactions.

Micro calls and silence are both acoustic behaviors that make bats much less acoustically conspicuous to conspecifics. Micro calls are extremely low intensity, high frequency and short duration—all features that make them difficult to detect by eavesdroppers but that appear to provide some minimum amount of sensory ability to avoid damaging collisions with objects such as trees^14^. It is not currently known why bats switch between silence and producing micro calls. Playbacks of high-intensity hoary bat echolocation calls are highly attractive to other hoary bats and aggressive chasing events are common in our study area^14^. These data indicate that micro calls and silence may be stealth behaviors aimed at avoiding detection by conspecifics. Bats “turn on” high-intensity echolocation during chases, either after they have been discovered or after they have revealed their position by engaging in close pursuit of another bat (See Fig. 4d in Corcoran and Weller, 2018^14^).

It is noteworthy that all the bats in the current study were male and all the bats in the earlier study^14^ for which sex could be determined were also male. Also, both studies were conducted during autumn when mating occurs. It is not currently known if silence and micro call are used by females or at other times of year. Given use by males engaged in social interactions during the mating season we posit that these echolocation behaviors are involved in male-male competition.

In contrast to social interactions, feeding buzzes were highly correlated with periods of time when bats were using high-intensity echolocation (Fig. 2). Feeding buzzes were always preceded by high-intensity search phase echolocation. These data support the hypothesis that bats require high-intensity echolocation for detecting insect prey and that they are unable to detect insects at sufficient distance for prey capture using either micro calls^14^ or vision alone^18^. The data also contradict the hypothesis (H_2_) that hoary bats use micro-calls or silence as a feeding strategy. In summary, hoary bats appear to switch sensory strategies for different tasks—using high-intensity echolocation for commuting and feeding and micro calls and silence during periods when they are engaged in social activities and seek to avoid detection by conspecifics.

### Hoary bats fly in silence for extended periods of time

We documented 155 cases of bats flying for at least 5 s without producing detectable echolocation calls, including 122 recordings where bats were apparently silent for the full 10-s recording. This was 400% of the longest interval between calls recorded when bats were using high-intensity echolocation (Fig. S1).

Calibration of the on-board microphones indicated that they should reliably detect calls produced with a source level of at least 75 dB SPL at 10 cm (Supplementary Information). Could hoary bats be producing calls for echolocation that microphones were unable to detect? A call produced at 75 dB or lower would allow hoary bats to detect a large obstacle such as a tree only at 1.5 m^19^. It was shown in a previous study^14^ that hoary bats were unable to avoid colliding with a mist net when initiating an avoidance maneuver at 2–3 m. Thus, the 1.5 m warning distance afforded by a 75 dB call would be insufficient even for avoiding collisions with the largest of obstacles. We argue that a call at a level below what our microphones were capable of detecting would serve no known sensory function at the high flight speeds exhibited by hoary bats. While it is possible that bats could produce sounds < 75 dB for purposes such as communication, these data strongly suggest that the bats in our study flew for extended periods of time without using echolocation. This is in contrast with micro calls, which are produced at higher intensities (83–109 dB) and would provide bats some minimal sensory ability^14^.

Bats were observed flying in apparent silence in most or all of recordings made for periods of 20–30 min (Fig. 1). Note that we recorded echolocation for 10 s every 3 min (night 1) or 10 s every minute (night 2). Therefore, we cannot conclude that bats were silent this entire time. Regardless, these data show some bats regularly employ silence as a sensory strategy for extended periods of time in flight. This demonstrates that bats are much less reliant on echolocation than previously known.

### Flying without echolocation is the exception, not the rule

A previous study documented hoary bats using high-intensity echolocation in only 6 of 79 (7.6%) flights over microphone arrays^14^. Therefore, we predicted that hoary bats would use high-intensity echolocation rarely. However, in the current study bats used high-intensity echolocation in 75.6% of recordings made using on-board tags (including feeding buzzes where high-intensity echolocation predominated; Table 1). The main difference between the two studies is that the earlier study documented hoary bat activity at specific locations–riparian corridors—whereas the current study followed individual bats for up to two nights as they presumably moved through a variety of locations. The riparian corridors used in the earlier study were the site of frequent social interactions among hoary bats and it was proposed that hoary bats were engaged in mating-related behavior at these locations^14^. Therefore, hoary bats appear to use high-intensity echolocation most of the time that they are flying but switch to using micro calls and silence for extended periods in certain situations where they aim to avoid detection by other bats.

**Table 1:** Categorization of acoustic behaviors for 10 male hoary bats (*Lasiurus cinereus*) recorded using on-board ultrasonic recording devices.

Bat ID	N	High intensity	Micro	Silent	Buzz	Social	Other bats
29507	84	84; **100.0%**	0; **0.0%**	0; **0.0%**	0; **0.0%**	0; **0.0%**	1; **1.2%**
2A507	6	6; **100.0%**	0; **0.0%**	0; **0.0%**	0; **0.0%**	0; **0.0%**	0; **0.0%**
2B500	256	237; **92.6%**	0; **0.0%**	3; **1.2%**	11; **4.3%**	5; **2.0%**	9; **3.5%**
2B501	88	82; **93.2%**	0; **0.0%**	0; **0.0%**	5; **5.7%**	1; **1.1%**	2; **2.3%**
2B502	187	90; **48.1%**	20; **10.7%**	56; **29.9%**	4; **2.1%**	17; **9.1%**	23; **12.3%**
2C502	134	42; **31.3%**	32; **23.9%**	49; **36.6%**	9; **6.7%**	2; **1.5%**	3; **2.2%**
2C507	6	4; **66.7%**	0; **0.0%**	2; **33.3%**	0; **0.0%**	0; **0.0%**	0; **0.0%**
52501	70	49; **70.0%**	2; **2.9%**	14; **20.0%**	1; **1.4%**	4; **5.7%**	4; **5.7%**
5E500	140	102; **72.9%**	0; **0.0%**	31; **22.1%**	3; **2.1%**	4; **2.9%**	25; **17.9%**
5F507	26	25; **96.2%**	1; **3.8%**	0; **0.0%**	0; **0.0%**	0; **0.0%**	0; **0.0%**
Total	997	721; **72.3%**	55; **5.5%**	155; **15.5%**	33; **3.3%**	33; **3.3%**	67; **6.7%**

N indicates total number of recordings where it was determined the bat was flying. Data show number and percent of 10-s audio files with high-intensity or micro calls, no detected echolocation (“silent”), feeding buzzes (“buzz”), social interactions indicative of chases (“social”) and echolocation calls recorded from other bats (“Other bats”). Numbers of recordings are shown in normal type, percentages in bold.

### Hoary bats exhibit higher flight maneuvering when using reduced echolocation

Hoary bats exhibited lowest flight maneuvering when producing high-intensity echolocation calls at low calling rates (Fig. 3B), which are typically used by bats flying in open space. Higher rates of flight maneuvering occurred during all other acoustic behaviors, including when bats flew in silence and produced micro calls (Fig. 3B). These data provide strong evidence that silence and micro calls are not employed during straight commuting flights in the open. Thus, hoary bats are not shutting off their echolocation simply because they don’t need it when they are flying far from obstacles such as terrain or vegetation^20,21^. Instead, hoary bats have been observed to use silence and micro calls when flying close to the ground and background vegetation, reacting as necessary by turning on their high-intensity echolocation when they detect obstacles or other bats^14^.

This raises the question of what senses hoary bats use when not using echolocation. There is increasing evidence that bats complement echolocation with vision^18,22,23^ and spatial memory^24,25^. The current data do not allow us to determine to what extent hoary bats are using these sensory modalities in flight. However, natural variation in the use of echolocation in hoary bats may allow further exploration of the benefits and limitations of different sensory modalities in bats.

## Conclusions and implications

The results of this study indicate that bats use high-intensity echolocation most of the time but switch to using micro calls and silence in specific situations to avoid eavesdropping by other bats. One important implication of this research is for understanding bat collisions with wind turbines, as hoary bats are the most frequently killed bat species at wind energy facilities in North America^26^. Our results indicate that bats are unlikely to collide with wind turbines simply because they are commuting without using echolocation^20,21^. However, it is possible that hoary bats (and potentially other species) aggregate near wind turbines and use micro calls and silence as they engage in social behaviors around them. It has been proposed that wind turbines may serve as social aggregation sites because they are large structures that could be detected visually from a distance^27,28^. Additional studies are required to test the social aggregation hypothesis. For example, a combination of 3-D thermal videography and synchronous ultrasound recordings could be used to document bat flight tracks and echolocation behavior^29^.

Our data provide the most direct evidence to date that bats fly for extended periods of time without using echolocation. Although bats were once considered almost exclusively reliant on echolocation, it is now clear that bats also incorporate other senses for nocturnal navigation^18,22,30,31^. Studies of species that switch echolocation strategies and the conditions under which they do so will continue to improve our understanding of the range of sensory modalities employed by these fascinating animals.

Our data indicate that hoary bats reduce their use of echolocation to avoid detection by conspecifics. We suspect this may be mating-related behavior, because our study occurred during the autumn when male hoary bats show signs of mating readiness^15^ and social interactions among hoary bats have been observed at our field site. However, the detailed mating behavior of hoary bats remains largely unknown, especially compared to other aspects of their biology such as feeding and roosting^32^. Additional research is needed to determine whether the stealth behaviors documented here serve a mating function—either between male competitors or between potential mates. However, our findings indicate that selective pressure from social interactions appear to have driven bats to abandon, at least temporarily, one of the very adaptations that allow them to dominate night skies.

## Methods

Bat capture, handling, and tag attachment were carried out in accordance with guidelines of American Society of Mammologists^33^ under permit from the California Department of Fish and Wildlife (# SC-002911). Experimental methods were approved by the Institutional Animal Care and Use Committee of the USDA Forest Service (IACUC 2017-014). We captured bats using 2.6-m high mist nets in a triple-high configuration. We measured forearm length and mass and determined species, age, sex, and reproductive status for each captured individual.

We used Vesper Pipistrelle on-board audio-recording devices with an accelerometer (ASD Tech, Haifa Israel) to quantify bat movement throughout the duration of attachment. We used the smallest possible battery (0.5 g) which was sufficient to allow a 3-h recording period on the first night and up to a 4-h recording period on the second night. Tags were programmed to record for 10 s once every 3 min from 23:00 to 02:00 on the first night and for 10 s once every minute from 19:00 to 23:00 on the second night. We recovered tags from bats tagged between September 28th and October 7th. Sunset was at 19:02 on September 29th and 18:47 on October 8th. Unfortunately, the timing mechanism on the tags malfunctioned some of the time, causing only some of the recordings to have synchronous audio and accelerometer data (See Results).

We attached Holohil LB-2X VHF transmitter (0.27 g) to the audio tags so we could locate the device once it detached from the bats. We coated the entire tag package (except the microphone opening) with liquid silicone followed by a latex sleeve covering to provide protection from the environment. The total tag package had a mass of 2.9 g which represented 10.6–12.5% of the mass of the bat. Several studies conducted in flight tents and in the field have shown no adverse consequences of payloads up to 15% for short duration deployments^16,34^. The diversity of natural behaviors that we observed, including prey pursuit, conspecific interaction, and extended flight over multiple nights indicates that hoary bats are capable fliers with this payload, however we cannot rule out the possibility that tags altered the behaviors that were observed.

We attached tags to the posterior dorsum of bats using latex surgical adhesive (Torbot Liquid Bonding Cement, Torbot Group Inc. Cranston, Rhode Island). We used the minimum quantity of adhesive that we estimated would be necessary for tags to remain affixed to bats for 2 nights. We recovered tags by using ground- and aircraft-based VHF telemetry to determine the general location of the shed tag, followed by homing in on the VHF signal using ground-based telemetry. Final recovery of tags was achieved using visual searches of the ground.

### Microphone calibration

We calibrated on-board microphones to determine the minimum sound pressure level (SPL) at which we could reliably detect micro calls. We did this by broadcasting a series of micro calls from an Avisoft (Glienicke/Nordbahn, Germany) Scanspeak ultrasonic speaker to the on-board tags. The series of micro calls consisted of a single high-quality micro call that was broadcast 30 times with each successive call being 3 dB lower in SPL. The absolute intensity of the broadcast was calibrated by broadcasting the same signal to a G.R.A.S (Holte, Denmark) 40DP 1/8″ microphone, which itself was calibrated with a G.R.A.S 42AB sound calibrator. For both the calibration of the sound playback and the broadcasts to the on-board microphone, the microphones were placed 10 cm from the speaker. We repeated this procedure three times for each of three microphones that had been recovered from the bats and determined the SPL of the lowest amplitude micro call that could be detected on all nine broadcasts. This SPL was used as the minimum detectable level at which our microphones could detect micro calls.

### Data processing

#### Determining whether bats are flying

We used custom MATLAB (Natick, MA) scripts to analyze ultrasound and accelerometer recordings. We first determined whether bats were in flight for each recording. Unfortunately, we were only able to record simultaneous accelerometer and acoustic data for 364 out of 2241 recordings. For these recordings, we independently classified each file as flight or no flight using only the accelerometer data and only the audio data. Accelerometer recordings showed clear and prominent wingbeat oscillations in the dorsoventral, or Z-axis (Fig. S2A). One observer used a custom program (AccelVis) to visualize and manually classify all accelerometer files. We also quantified the magnitude of wingbeat oscillations by measuring the root-mean-square magnitude of signals after applying a high-pass filter of 4 Hz (Bats used wingbeat frequencies of approximately 8 Hz).

A different observer classified all audio recordings as flight or no flight based on the presence or absence of low-frequency wind noise generated by the relative motion of the bats flying through the air (Fig. S2). The Individuals conducting the audio and acceleration analyses were blind to one another’s data. As with the accelerometer data, we analyzed all files both qualitatively and quantitatively. For the qualitative analysis, a user visualized files using a custom program (AudioBrowser; available with all data files as supplementary data) and noted presence or absence of low-frequency wind noise. We also quantified this wind noise by measuring the RMS magnitude of signals after applying a 1-Hz low pass filter. This resulted in a distinct bimodal distribution of low frequency magnitudes that corresponded to no wind and wind conditions with the two peaks being separated by approximately 30 dB. A small number of files (< 10%) had values between the two peaks because of abrupt noise bursts from an unknown origin. This noise had a distinct spectral-temporal profile and all files containing this noise were classified manually.

We compared the classification of flight/no-flight from accelerometer and audio data (N = 364 files) and found 100% correspondence. Therefore, for audio files lacking synchronous accelerometer data, we used low-frequency wind noise to determine flight *vs.* no flight conditions.

#### Quantifying flight maneuvering

For each accelerometer recording, we quantified the magnitude of flight maneuvering (Fig. 3) using the lateral and anterior–posterior components of the accelerometer measurements. We first detrended each signal by subtracting the mean signal value. We next applied a 4 Hz low-pass filter to each signal to remove wingbeat frequency oscillations and highlight maneuvers lasting more than one wingbeat. Finally, we took the root-mean-square value of the filtered signals as the overall measure of flight maneuvering.

#### Classifying acoustic behaviors

We manually classified each 10-s acoustic recording as either silence, micro calls, high-intensity calls, feeding buzz or social interaction (Fig. 1). Recordings containing a feeding buzz or social interaction were classified as such (see below for how these events were identified). Otherwise, recordings were classified as silence, micro calls or high-intensity calls based on the call type that occupied the majority of the recording (> 5 s). This 5 s threshold is twice the longest pulse interval recorded for echolocation calls (Supplementary Information), and therefore represents a conservative threshold for identifying silent periods.

High-intensity calls could be identified by their consistently high signal levels. For recordings where no calls were initially detected, the observer made a second examination of the recording using a custom 55–90 kHz bandpass filter setting that highlights micro calls (Fig. 1D). A second observer also examined all files where either no calls or micro calls were detected by the first observer to confirm classification. Recordings were processed both by visualization of spectrograms and by listening to slowed-down recordings through headphones.

Hoary bat feeding buzzes have a characteristic pattern involving a rapid increase in calling rate, and progressively decreasing call intensity (Fig. 1B)^35,36^. In contrast, social interactions involve prolonged (often several seconds) high-intensity echolocation calls produced at a high rate (e.g., 50–100 Hz) with a second bat also producing echolocation calls at a relatively high calling rate^14^. Echolocation calls of “other” bats (which could be present in any of the recordings) could be distinguished from the calls of the bat with the tag because they were typically recorded at a much lower intensity levels that increased and decreased, presumably as the other bat approached and then withdrew from the focal bat and were temporally out of phase with calling rate of the tagged bat. Calls classified as “other bat” also had lower calling rates compared to social interactions.

#### Statistical analysis

Acoustic recordings were organized by individual bat (Table 1) and by time of night (Fig. 2). To determine if bats exhibited consistent differences in the use of high-intensity echolocation, we measured the proportion of recordings including high-intensity echolocation for each bat night. Initial analysis of the data indicated that bats produced high-intensity echolocation during either most or all of the recordings (96–100%, including feeding buzzes) or at a considerably lower rate (< 80%). We used a one-sided binomial probability test to determine the likelihood of all five bats with two nights of data using high (> 96%) or low (< 80%) rates of high-intensity echolocation on each of the two nights they were recorded.

We used a permutation test to determine whether feeding buzzes and social calls were produced during periods when bats were producing fewer or more high-intensity echolocation calls than expected by chance. For this test, we kept the timing of recordings constant, but randomized the acoustic classifications shown in Fig. 2. For each feeding buzz and social call, we identified all recordings that occurred within 15 min and determined the proportion of those recordings that included high-intensity echolocation calls. This was iterated 10,000 times to generate a random distribution of the percent of recordings within 15 min having high-intensity echolocation calls (See Fig. 2B for an example randomization). The actual percent of recordings within 15 min containing high-intensity echolocation calls was then compared to this distribution to determine a *P* value.

We used linear regression to determine if cloud cover or moon illumination was correlated with the proportion of time bats used high-intensity echolocation calls. For each recording night, we used moon calendars to look up percent moon illumination. We also confirmed that the moon was up during the recording period for each night. We downloaded weather data for the weather station closest to our release site (Fortuna Rohnerville Airport, KFOT) and measured the proportion of time during our recording period each night when the cloud cover was either “overcast” or “mostly cloudy” *versus* “clear” or “partly clear”. We then conducted a linear regression to determine if there was a relationship between the proportion of recordings with high-intensity calls and either our nightly measure of moon illumination or cloud cover. We did not attempt to conduct an analysis of calling behavior and cloud cover at a finer temporal resolution (such as hourly) to avoid temporal autocorrelation that might confound our analysis.

Finally, we compared our measures of flight maneuvering across recordings that were classified as different acoustic behaviors (Fig. 3D). Because of the highly skewed distribution, we used a non-parametric Kruskal–Wallis test with Tukey post-hoc comparisons to determine whether median levels of flight maneuvering differed between recordings classified as different acoustic behaviors.

## Supplementary Information

Supplementary Information 1.

Supplementary Audio 1.

Supplementary Audio 2.

Supplementary Audio 3.

Supplementary Audio 4.

## Data Availability

All processed data and computer code for generating figures as well as all original audio and accelerometer files, and custom software for their visualization and analysis (compiled for use on Windows operating systems without need for any licenses) are available as a Dryad data depository (https://datadryad.org/stash/share/b81qQeg9hh74c3t0XsAxdqXJjQrXxdx5D_bhrcWMN6Y).
