# Supplementary material for: Silence and reduced echolocation during flight are associated with social behaviors in male hoary bats (Lasiurus cinereus)
Source: Sci Rep. 2021 Sep 20;11:18637. doi: 10.1038/s41598-021-97628-2 (PMC8452715; doi:10.1038/s41598-021-97628-2)
Supplement: Supplementary file 1 — Supplementary Information 1. [file 41598_2021_97628_MOESM1_ESM.docx]

# Silence and reduced echolocation during flight are associated with social behaviors in male hoary bats (*Lasiurus cinereus*)

Aaron J. Corcoran^1*†^, Theodore J. Weller^2†^ , Annalise Hopkins^1^ and Yossi Yovel^3^

^1^University of Colorado, Colorado Springs, Department of Biology, 1420 Austin Bluffs Blvd, Colorado Springs, CO 80918 USA

^2^USDA Forest Service, Pacific Southwest Research Station, 1700 Bayview Dr., Arcata, CA 95521 USA

^3^Tel Aviv University, School of Zoology, P.O. Box 39040, Tel Aviv 6997801, Israel

* Correspondence: acorcora@uccs.edu

^†^ These authors contributed equally to this manuscript

# Supplementary Materials

Supplementary Information

Figures S1, S2

Supplementary Audio Files 1-4

# Supplementary Information

## Echolocation pulse intervals

We analyzed a random sample of 200 of the 721 recordings with high-intensity echolocation calls (excluding feeding buzzes) to determine typical hoary bat calling rates, as indicated by the time interval between successive calls or pulse interval (from beginning of one call to beginning of next call). This resulted in 4019 pulse intervals, which ranged from 30.7 to 2544 ms (Fig. S1). Pulse intervals had distinct peaks at intervals of approximately 1/8^th^ of a second (0.125 s), which corresponded to observed wingbeat frequencies (7.8 wingbeats/s overall; 7.5-8.3 wingbeats/s range across individuals based on accelerometer data). Hoary bats used a wide range of pulse intervals, corresponding to anywhere between several calls per wing beat (pulse intervals < 125 ms) to 1 call every 20 wingbeats (pulse interval = 2.5 s). Nonetheless, our recording period of 10 s was approximately 4 times longer than the maximum recorded pulse interval, which indicates that recordings lacking echolocation calls were not a result of long intervals between calls. Based on this analysis, we conservatively used a threshold of 5 s for identifying recordings with periods of silence.

## Individual variation in echolocation behavior

Individuals (all male) showed notably different patterns of acoustic behaviors (Table 1; Figure 2). Note that two individuals (2A507 and 2C507) only had 6 recordings a piece, and therefore they were not considered in our analysis of individual variation in echolocation behavior. Four individuals used high-intensity echolocation most or all the time (92.6-100%); while the other four individuals used high-intensity echolocation less often (31.3-72.9%). Individuals varied in the proportion of recordings having feeding buzzes (0.0-6.7%), social interactions (0.0-9.1%) and echolocation calls from other bats present (0.0-17.9%). However, none of these behaviors correlated with proportion of time bats used high-intensity echolocation (linear regression; F = 0.55-0.11; P > 0.05).

Broad patterns of echolocation behavior were consistent night-to-night for the five bats with two nights of data (Fig. 2). Two bats (2B501 and 2B500) used high-intensity echolocation (including feeding buzzes) throughout most or all of recordings on both nights (95.3-100%), while the remaining three bats (5E500, 2C502 and 2B502) exhibited lower rates of high-intensity echolocation (27.5-81.9% of recordings) on both nights. This finding was statistically significant (binomial probability test; P = 0.031), indicating only some individuals make frequent use of inconspicuous echolocation behaviors.

These data indicate that individual identity may be a better predictor of echolocation behavior than factors related to a particular night, such as cloud cover or moon illumination. This could result from individuals being in different behavioral states. For example, some individuals could be more motivated to migrate, while others may be more motivated to seek mating opportunities. Alternatively, it could reflect males that are using different reproductive strategies or have different social status, with some choosing more stealthy tactics than others. We found no obvious patterns in echolocation strategies employed by individuals as a function of forearm length, mass, or reproductive status of the bat as assessed prior to tag attachment. Additional studies are needed to determine the specific biological function of micro calls and silence in hoary bats, and the characteristics of individuals that employ them.

## Calibration of microphones and modeling of echolocation emissions indicates that hoary bats fly in silence

Calibration of the on-board microphones indicates that they can detect micro calls produced at 55 dB peak equivalent sound pressure level re. 20 µP (peSPL; 10 cm reference distance). We assume an additional 20 dB attenuation from the tags being placed nearly 180 degrees behind the direction of sound emission. This is a conservative value compared to a previous study ^1^ and in line with calibrations using the same microphone tag and another bat species (*Parastrellus kuhlii*; Y. Yovel, *Unpublished data*). Signal attenuation for on-board microphones is independent of frequency ^1^, and therefore, these calibration data should apply to hoary bat micro calls. This indicates that our tags should have picked up any calls with a source level of at least 75 dB. It was previously shown that micro calls have a median source level of 99.8 dB and that the quietest micro calls detected were 81.3 dB ^2^. Therefore, our microphones should be capable of detecting even the quietest micro calls.

Figure S1. Histograms of pulse intervals from high-intensity echolocation calls made by hoary bats shown as total time (top) and number of calls made (bottom). A smoothing spline is shown in red to highlight peaks in the data.


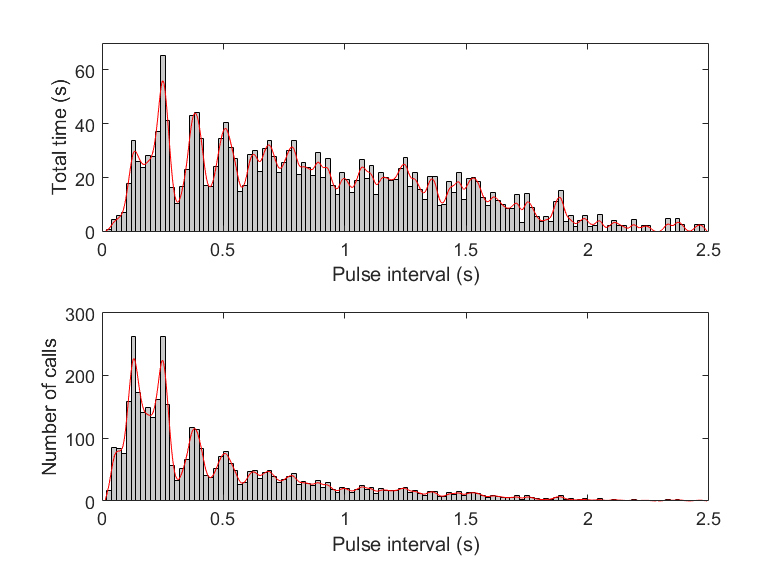


Figure S2. Example acceleration (A, B) and ultrasonic recordings (C-F) of hoary bats flying (A, C, E) or not flying (B, D, F) when no echolocation calls were detected. Audio recordings show distinct wingbeat oscillations at approximately 8 Hz during flying trials (C, E), but these oscillations are absent when bats are not flying (D, F). The red line in (C) indicates the sound envelope using a 20 Hz low-pass filter. High-amplitude sound oscillations also occur at approximately 8 Hz during flight (C). This sound is predominantly at low frequencies (< 5 kHz) as shown in the spectrogram in E. The low-level periodic sound in D, F is an artefact of the recording device.
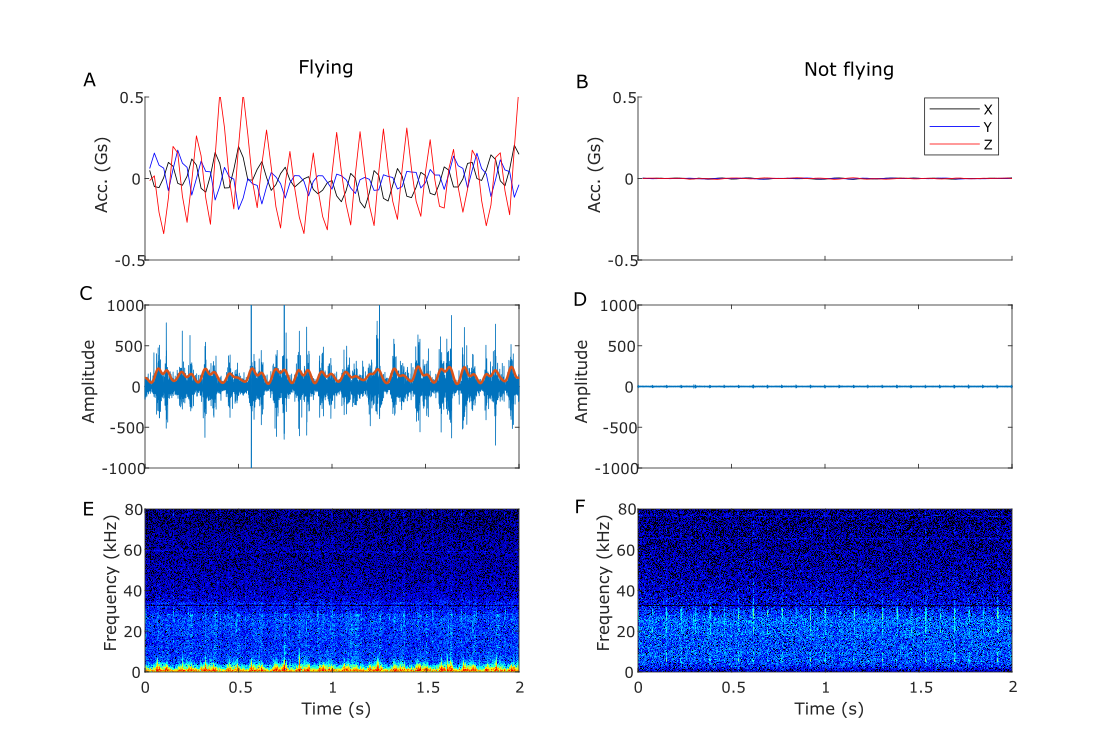


## Supplementary References

1. Stidsholt, L. *et al.* A 2.6‐g sound and movement tag for studying the acoustic scene and kinematics of echolocating bats. *Methods Ecol. Evol.* **10**, 48–58 (2019).

2. Corcoran, A. J. & Weller, T. J. Inconspicuous echolocation in hoary bats (Lasiurus cinereus). *Proc. R. Soc. B Biol. Sci.* **285**, 20180441 (2018).

Supplementary Audio File 1. Example recording of hoary bat high-intensity echolocation calls. Audio is played back at 20% of normal speed.

Supplementary Audio File 2. Example recording of hoary bat feeding buzz. Audio is played back at 20% of normal speed.

Supplementary Audio File 3. Example recording of hoary bat social interaction. Audio is played back at 20% of normal speed.

Supplementary Audio File 4. Example recording of hoary bat flying in apparent silence. Note the oscillations of wind noise caused by the wing beats. Audio is played back at 20% of normal speed.
